# Probiotics Ameliorate Atrial Fibrillation-Associated Biomarkers and Inflammation in Rats via Modulation of the Gut Microbiota and NLRP3 Inflammasome Pathway

**DOI:** 10.5812/ijpr-168612

**Published:** 2026-04-18

**Authors:** Yeran Zhu, Long Bai, Ruibin Li, Yichen Li, Hongxia Hou, Jidong Zhang

**Affiliations:** 1 Affiliated Hospital of Hebei University, Baoding, China; 2 The Second Hospital of Hebei Medical University, Shijiazhuang, China

**Keywords:** Atrial Fibrillation, Probiotics, Intestinal Microbiota, NLRP3 Inflammasome Signaling Pathway

## Abstract

**Background:**

The gut-heart axis, particularly the role of gut microbiota and the NOD-like receptor family pyrin domain containing 3 (NLRP3) inflammasome, is increasingly implicated in the pathogenesis of atrial fibrillation (AF). While probiotics show therapeutic potential, their mechanism in AF remains unclear.

**Objectives:**

This study investigates whether probiotics mitigate AF pathology by restoring gut microbiota homeostasis and inhibiting NLRP3 activation.

**Methods:**

An AF model was induced in rats via acetylcholine (ACH)/CaCl₂ infusion. Animals were divided into five groups: Sham, AF, AF+Probiotics, AF+MCC950 (NLRP3 inhibitor), and AF+Probiotics+MCC950. We analyzed gut microbiota (16S rRNA sequencing), serum short-chain fatty acids (SCFAs; GC-MS), and the expression of NLRP3 pathway components [NLRP3, caspase-1, interleukin-1β (IL-1β)] and AF biomarkers [pentraxin 3 (PTX3), interleukin-6 (IL-6), tumor necrosis factor-alpha (TNF-α), chemerin, galectin-3 (Gal-3)] via immunohistochemistry (IHC), Western blot, and real-time quantitative polymerase chain reaction (RT-qPCR).

**Results:**

Atrial fibrillation rats exhibited gut dysbiosis, characterized by reduced diversity (Shannon index significantly decreased vs. Sham, P < 0.01), decreased beneficial bacteria (e.g., Lactobacillus), and lower SCFA levels (acetic, propionic, and butyric acids reduced by 40 - 50%, P < 0.05). Probiotics restored microbial diversity, increased SCFA production (to levels comparable to Sham, P < 0.05), and suppressed NLRP3 inflammasome activation, as evidenced by reduced levels of NLRP3, caspase-1, and IL-1β (protein and mRNA expression decreased by 50 - 70%, P < 0.01). Consequently, the expression of key AF biomarkers was significantly downregulated (PTX3, IL-6, TNF-α, chemerin, Gal-3 reduced by 40 - 60%, P < 0.05). The combined intervention of probiotics and MCC950 yielded the most pronounced effects (all markers further reduced vs. either treatment alone, P < 0.05).

**Conclusions:**

Probiotics alleviate AF-related inflammation and biomarker expression, potentially by modulating the gut microbiota and inhibiting the NLRP3 inflammasome pathway. These findings highlight the gut-heart axis as a promising target for AF management.

## 1. Background

Atrial fibrillation (AF) is a rapid supraventricular arrhythmia characterized by disorganized atrial electrical activation and ineffective atrial contractions. Research indicates that approximately 30 million individuals worldwide are affected by AF, with its prevalence showing a strong positive correlation with age. As the population ages, the prevalence of AF rises significantly, increasing from 0.5% in individuals aged 50 - 59 years to nearly 9% in those aged 80 - 89 years (1). Notably, some patients with AF exhibit atypical symptoms or remain asymptomatic, contributing to a high rate of underdiagnosis in clinical settings. Furthermore, AF is associated with various complications, including stroke, heart failure, renal dysfunction, cognitive impairment, and increased mortality, thereby imposing a substantial economic burden on global healthcare systems (2, 3). Current treatment options for AF primarily consist of catheter ablation, ventricular rate control medications, and anticoagulation therapy. However, these treatments have notable limitations, such as high risks of side effects, incomplete therapeutic efficacy, high recurrence rates, poor patient adherence, and improper medication use, all of which may lead to drug-induced arrhythmias (4). Consequently, there is an urgent need for further research into effective AF treatment strategies.

The gut microbiome comprises the microorganisms that inhabit the intestinal tract and coexist symbiotically with the host, forming a natural barrier in conjunction with the intestinal mucosa to maintain human health (5). Through long-term evolution, the gut microbiome and the host have established a stable symbiotic relationship characterized by mutual restraint and interdependence, maintaining a dynamic balance (6). As research into the relationship between the gut microbiome and host health expands, an increasing number of studies have demonstrated a close association between the gut microbiome and cardiovascular diseases, including AF (7). The study indicated that AF patients exhibit reduced Enterobacteriaceae levels while showing increased *Parabacteroides*, *Clostridium*, *Streptococcus*, and *Ruminococcus populations* (8). Another study suggested that metabolites derived from the gut microbiome could exacerbate inflammatory responses and myocardial fibrosis, activate the cardiac autonomic nervous system, and thereby promote AF progression (9). Consequently, research targeting the gut microbiome has emerged as a critical focus in the diagnosis and treatment of related diseases (10).

When intestinal flora imbalance occurs, the levels of pathogen-associated molecular patterns in the gut increase. Upon recognition by pattern recognition receptors, this triggers a cascade of pro-inflammatory responses. Among these, the classical NOD-like receptor family pyrin domain containing 3 (NLRP3) inflammasome signaling pathway has been identified as a key mechanism contributing to AF development (11, 12). The NLRP3 inflammasome, one of the most characteristic inflammasomes, is a multi-protein complex consisting of the cytoplasmic innate immune receptor NLRP3, the adaptor protein apoptosis-associated speck-like protein containing a caspase recruitment domain (ASC), and the effector enzyme caspase-1. This complex recognizes a wide range of microbial or danger signals and mediates downstream inflammatory responses (13). Therefore, targeting the NLRP3 inflammasome may represent a promising therapeutic strategy for AF.

Probiotics, as a novel form of intestinal microecological therapy, play a pivotal role in improving intestinal microbiota dysbiosis, enhancing intestinal barrier function, and reconstructing a microecological structure conducive to host health (14). In addition, probiotics can prevent and treat various diseases by modulating the immune system and exerting anti-inflammatory effects. Research has demonstrated that the consumption of active lactic acid bacteria suppresses the overactivation of the NLRP3 inflammasome and downstream caspase-1 pathway in macrophages, thereby alleviating ulcerative colitis (15). Probiotics improve IgA nephropathy by restoring intestinal microbiota homeostasis and attenuating NLRP3 signaling (16). However, whether probiotics influence AF through regulation of the gut microbiota and modulation of the NLRP3 signaling pathway, as well as the underlying mechanisms, remains to be elucidated.

Despite evidence linking gut dysbiosis and NLRP3-driven inflammation to AF, the therapeutic potential of probiotics and the underlying mechanisms remain largely unexplored.

## 2. Objectives

this study aims to investigate the effects of a specific probiotic intervention on AF pathology, focusing on its role in rectifying gut microbiota imbalance, regulating microbial metabolites (SCFAs), and subsequently suppressing the NLRP3 inflammasome signaling pathway. Our work seeks to provide preclinical evidence for a novel gut-centric approach to managing AF.

## 3. Methods

### 3.1. Atrial Fibrillation Model Construction and Drug Administration

Male SD rats (8 weeks old) were purchased from the Hubei Provincial Laboratory Animal Research Center. All animal experiments and procedures were approved by the Institutional Animal Care and Use Committee of Hebei University (Ethics Number: HBU2024R052). After a 7-day adaptive feeding period, the rats were randomly divided into five groups (n = 5 per group): Sham, AF, AF+Probiotics, AF+MCC950, and AF+Probiotics+MCC950. Except for the Sham group, AF models were established in the remaining groups by injecting a freshly prepared mixture of acetylcholine (ACH, 66 μg/mL) and CaCl_2_ (10 mg/mL) intravenously at a dose of 0.1 mL/100 g (HY-B0282, MCE) for 7 consecutive days. Following AF model establishment, probiotics were administered via gavage for 14 days. The probiotic supplement (Flora-Focus^®^ BB-G95, Runying Bioengineering (Shanghai) Co., LTD) consisted of Bifidobacterium breve BB-G95, with a total dosage of 1×10^9^ colony-forming units (CFU) in 1 mL of saline per day. Concurrently, MCC950 (HY-12815, MCE) was injected intraperitoneally at a dose of 10 mg/kg once daily for 14 days. At the end of the treatment period, rats were anesthetized with 2.5% tribromoethanol (6 mL/kg, intraperitoneal injection). Under direct vision, the abdomen and chest were opened, and 5 - 8 mL of blood was collected from the heart. The blood samples were left at room temperature for 30 min and then centrifuged at 3500 r/min for 10 min. The supernatant serum was collected and stored at -80°C. Subsequently, the right auricle was incised, and 30 mL of physiological saline was perfused into the left ventricle until the liver turned yellowish-brown, indicating complete perfusion. Heart tissues were then sectioned transversely. The ventricular and part of the atrial tissues were fixed in 4% paraformaldehyde, while the remaining tissues were frozen for further analysis.

### 3.2. 16S rRNA Gene Sequencing

The DNA of fecal microbiota was isolated, and its purity and concentration were assessed using 1% agarose gel electrophoresis. The V3-V4 region of the 16S rRNA gene in rat feces was amplified by PCR with the forward primer 27F (5'-AGRGTTYGATYMTGGCTCAG-3') and the reverse primer 1492R (5'-RGYTACCTTGTTACGACTT-3'). The primers were synthesized by Shanghai Major Biomedical Technology Co., LTD. The purified PCR products were quantified using Qubit fluorometry, and high-throughput sequencing was conducted on the Pacbio sequencing platform by Shanghai Major Biomedical Technology Co., LTD.

### 3.3. SCFA analysis

The concentration of SCFAs in rat blood was measured using an Agilent 8890-5977B gas chromatography-mass spectrometry (GC-MS) system (Agilent Technologies, USA). Briefly, 100 μL of plasma was homogenized with 250 μL of 0.5% phosphoric acid solution. Following centrifugation at 13,000 rcf for 15 min at 4°C, 200 μL of the supernatant was transferred to a 1.5 mL centrifuge tube. Subsequently, 200 μL of n-butanol was added for extraction, and the mixture was centrifuged again at 13,000 rcf for 10 min at 4°C. The resulting supernatant was aspirated into a sample vial for GC-MS analysis. Data acquisition and processing were carried out using Masshunter quantitative software (Agilent, USA, version: v10.0.707.0).

### 3.4. Immunohistochemistry

Immunohistochemistry (IHC) staining was performed on paraffin-embedded heart tissue sections. Briefly, the sections were dewaxed and rehydrated, followed by antigen retrieval in 0.01 M Tris-EDTA buffer (pH 9.0) for 15 min. Subsequently, the sections were incubated with 3% hydrogen peroxide at room temperature for 15 min to block endogenous peroxidase activity. The tissue sections were then incubated overnight at 4°C with primary antibodies against interleukin-1β (IL-1β, 1:300, ab315084, Abcam), NLRP3 (1:200, DF7438, Affinity), and caspase-3 (1:200, AF5418, Affinity). On the following day, the sections were incubated with a goat anti-rabbit IgG (H + L)-HRP secondary antibody (1:500) at 37°C for 30 min. DAB staining was subsequently performed, followed by counterstaining with Mayer's hematoxylin, dehydration, air drying, and mounting. Images were captured using an optical microscope (Olympus BX53, magnification ×400). Quantification of IHC images was performed using ImageJ software (NIH, USA) by measuring the mean density of positively stained areas in five randomly selected fields per section.

### 3.5. Western Blot

Proteins were extracted from heart tissues, separated by 10% SDS-PAGE, and transferred onto polyvinylidene fluoride (PVDF) membranes. The membranes were blocked with 5% skim milk at room temperature for 1 h and subsequently incubated overnight at 4°C with the following primary antibodies: Anti-NLRP3 antibody (68102-1-lg, Proteintech), Anti-caspase-1 antibody (HA722222, Huane Bio), Anti-IL-1β antibody (26048-1-ap, Proteintech), Anti-pentraxin 3 (PTX3) antibody (DF8762, Affinity), Anti-IL-6 antibody (ab9324, abcam), Anti-tumor necrosis factor-alpha (TNF-α) antibody (bs-0549R, Bioss), Anti-chemerin antibody (833550-4-RR, Proteintech), Anti-Galectin-3 antibody (60207-1-Ig, Proteintech), and Anti-GAPDH antibody (AF7021, Affinity). The membranes were then incubated with goat anti-rabbit IgG (H+L)-HRP secondary antibody (1:5000, BA1054, BOSTER) at room temperature for 1 h, followed by washing. Protein expression was detected using an ECL kit (E411-04, Vazyme, Nanjing) and analyzed on a Bio-Rad imaging system.

### 3.6. Real-time Quantitative Polymerase Chain Reaction

Total RNA was extracted from rat atrial tissues using a Total RNA Extraction Kit (R1100, Solarbio, Beijing). cDNA was synthesized via reverse transcription using a cDNA Reverse Transcription Kit (QP056T, GeneCopeia, USA). mRNA expression levels were quantified by real-time PCR using a SYBR Green qPCR Master Mix (Vazyme Biotech, China). Primer sequences were designed according to the cDNA sequences retrieved from the GenBank database. GAPDH was used as the internal control gene for normalization. The relative mRNA expression levels were calculated using the 2^-△△Ct^ method. All primer sequences are listed below:

GAPDH: F-5′-TGTGAACGGATTTGGCCGTA-3′　R-5′-GATGGTGATGGGTTTCCCGT-3′ NLRP3: F-5′-CTGCATGCCGTATCTGGTTG-3′　R-5′-ATGTCCTGAGCCATGGAAGC-3′ Caspase-1: F-5′-GAAACGCCATGGCTGACAAG-3′　R-5′-ACATGATCGCACAGGTCTCG-3′ IL-1β: F-5′-GACTTCACCATGGAACCCGT-3′　R-5′-GGAGACTGCCCATTCTCGAC-3′ IL-6: F-5′-GAGACTTCCAGCCAGTTGCC-3′　R-5′-TGAAGTCTCCTCTCCGGACTT-3′ TNF-α: F-5′-ACTGAACTTCGGGGTGATCG-3′　R-5′-GCTTGGTGGTTTGCTACGAC-3′ PTX3: F-5′-ATTAAGGGCTTCGCTCCTGC-3′　R-5′-CTGGAGAGGCGAAGTTTGCT-3′ RARRES2（Chemerin）: F-5′-GGTGTGGACAGTGCTGATGA-3′　R-5′-CTGAGGCCCTTGCTTCAGTA-3′ Lgals3（Gal-3）: F-5′-CCTACGATATGCCCTTGC-3′　R-5′-CCCAGTTATTGTCCTGCTTC-3′

### 3.7. Statistical Analysis

All data were analyzed using GraphPad Prism software (version 9.4.0) and are presented as the mean ± standard deviation (SD). The normality of data distribution was assessed using the Shapiro-Wilk test. For comparisons between two groups, an unpaired two-tailed Student's *t*-test was used for normally distributed data, and the Mann-Whitney U test was used for non-normally distributed data. For comparisons among multiple groups, one-way analysis of variance (ANOVA) was performed if the data met the assumptions of normality and homogeneity of variance (verified by Brown-Forsythe test), followed by Tukey's post hoc test for multiple comparisons. If the data violated these assumptions, the non-parametric Kruskal-Wallis test was used, followed by Dunn's post hoc test. Differences in microbial community structure were assessed using non-metric multidimensional scaling (NMDS) based on Bray-Curtis distances. A P value of less than 0.05 was considered statistically significant.

## 4. Result

### 4.1. Probiotics Enhanced the Diversity and Composition of the Intestinal Microbiota, Thereby Inhibiting Atrial Fibrillation in Rats

**Figure 1.**
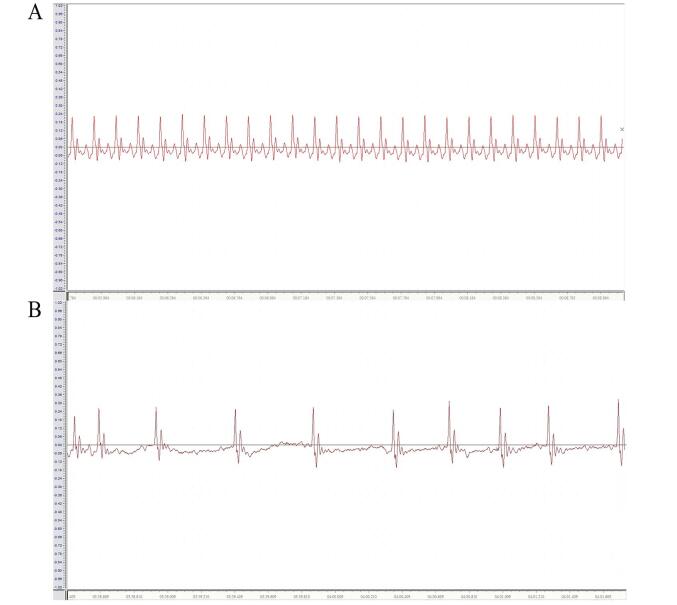
Electrocardiogram of rats. A, electrocardiogram of normal rats; B, electrocardiogram of atrial fibrillation (AF) rats.

**Figure 2.**
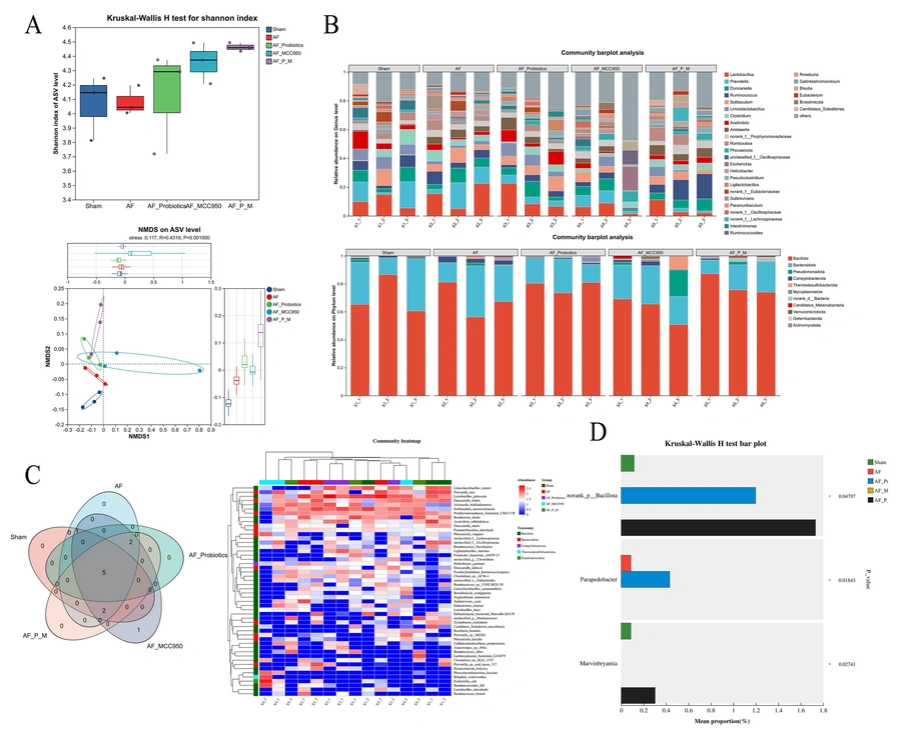
Probiotics enhanced the diversity and composition of the intestinal microbiota, thereby inhibiting atrial fibrillation (AF) in rats. A, the upper sub-plot shows the Kruskal-Wallis H test for the Shannon index. The lower sub-plot is an NMDS analysis at the ASV level (stress = 0.117, P = 0.001). B, community barplot analysis at the genus (upper) and phylum (lower) levels. C, the Venn diagram shows shared and unique ASVs, while the community heatmap clusters samples based on species abundance. D, Kruskal-Wallis H test bar plot validates significant differences in specific taxonomic units (e.g., norank_p_Bacillota, Parapedobacter) among groups.

After the AF model was successfully established in SD rats, typical AF electrocardiographic features (disappearance of P waves, irregular R-R intervals, and presence of small f waves) were observed, confirming the successful establishment of the AF model (Figure 1A and B). Subsequently, we employed 16S rRNA sequencing to analyze fecal samples from rats, assessing the richness and diversity of the intestinal microbiota to investigate the effects of probiotics on the gut microbiota in AF rats. The Shannon index, which quantifies microbial diversity, revealed that the intestinal microbiota diversity in the AF group was significantly reduced compared to the Sham group. In contrast, the AF+Probiotics, AF+MCC950, and AF+Probiotics+MCC950 groups exhibited significant improvements, with the AF+Probiotics+MCC950 group showing marked recovery. Additionally, NMDS analysis at the amplicon sequence variant (ASV) level (stress = 0.117, P = 0.001) demonstrated distinct clustering patterns between the AF and Sham groups, highlighting substantial structural differences in the microbiota. Conversely, the AF+Probiotics, AF+MCC950, and AF+Probiotics+MCC950 groups clustered closer to the Sham group, indicating restoration of the microbial community structure (Figure 2A). Notably, the combined treatment group (AF+Probiotics+MCC950) exhibited the closest clustering to the Sham group, suggesting a synergistic effect of probiotics and NLRP3 inhibition on microbiota recovery. Bar charts at the genus and phylum levels further elucidated compositional changes (Figure 2B): At the genus level, specific genera such as Lactobacillus displayed differential abundances across groups. At the phylum level, Bacillota and Bacteroidota exhibited group-specific variations, suggesting that probiotics modulated the relative abundance of key microbial taxa. A Venn diagram revealed that these five groups shared 5 ASVs. A community heatmap visually clustered samples based on species abundance, showing that Sham group samples tightly clustered together, while the AF group formed a distinct cluster. Notably, the AF+Probiotics, AF+MCC950, and AF+Probiotics+MCC950 intervention groups partially overlapped with the Sham cluster, reinforcing the recovery of microbial composition induced by the interventions (Figure 2C). Statistical validation using the Kruskal-Wallis H test confirmed significant differences in specific taxonomic units. For instance, the average proportion of norank_p_Bacillota was significantly higher in the AF+Probiotics+MCC950 group than in the Sham group (P = 0.04797), and the average proportion of Parapedobacter was significantly higher in the AF+Probiotics group than in the AF group (P = 0.01843). These findings underscore the regulatory effects of probiotics on specific intestinal microbial components (Figure 2D). Collectively, these results provide a comprehensive overview of the diversity, structural organization, and compositional characteristics of the intestinal microbiota, supporting the hypothesis that probiotics alter the gut microbiota.

### 4.2. Probiotics Treatment Increased the Metabolite Short-Chain Fatty Acids

**Figure 3.**
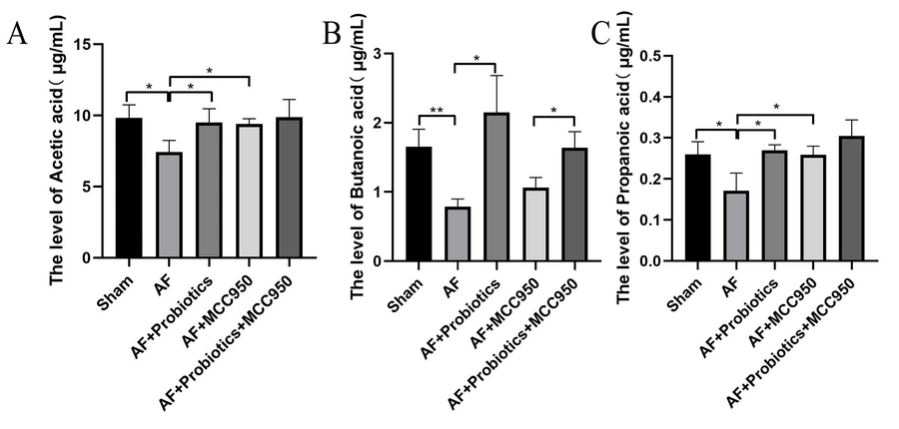
Probiotics treatment increased the metabolite short-chain fatty acids (SCFAs). A-C, gas chromatography-mass spectrometry (GC-MS) was used to measure SCFA concentrations (acetic acid, propionic acid, and butyric acid). * P < 0.05, ** P < 0.01.

Most of the bacteria with reduced relative abundance after AF model construction were closely associated with SCFA production. Subsequently, we used GC-MS analysis to quantitatively assess changes in blood SCFA levels. Compared with the Sham group, the levels of acetic acid, propionic acid, and butyric acid in the AF group were significantly decreased. In contrast, these SCFAs were significantly increased in the AF+Probiotics, AF+MCC950, and AF+Probiotics+MCC950 groups compared with the AF group, with some levels even surpassing those of the Sham group (Figure 3A, B, and C). Interestingly, butyric acid levels were significantly higher in the AF+Probiotics+MCC950 group than in the AF+MCC950 group, indicating that probiotics additively enhanced SCFA production beyond the effect of NLRP3 inhibition alone. However, no significant changes were observed in the levels of isovaleric acid, isocaproic acid, and caproic acid. Collectively, these findings indicate that the AF group was deficient in SCFA-producing bacteria, particularly those producing acetic acid, propionic acid, and butyric acid. Thus, probiotics regulated the generation of SCFAs by modulating the metabolic function of the intestinal flora.

### 4.3. Probiotics Inhibited the Activation of the NLRP3 Inflammasome Signaling Pathway

**Figure 4.**
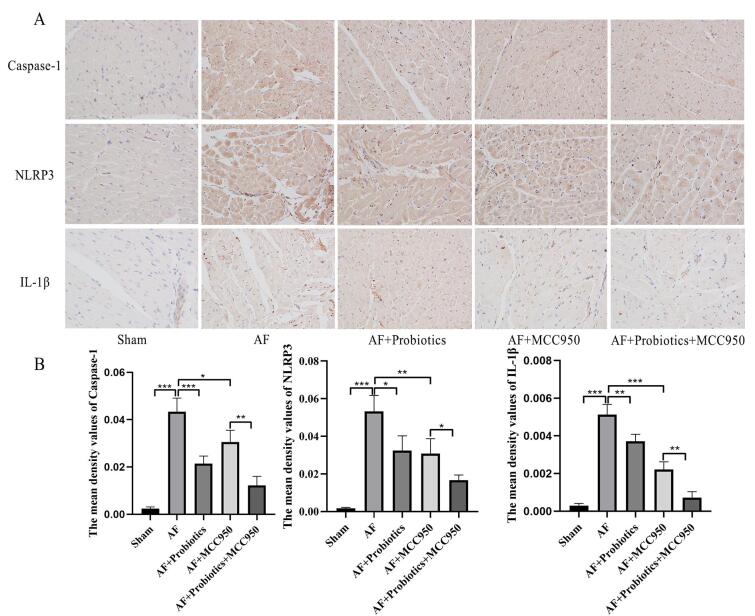
Probiotics inhibited the activation of the NOD-like receptor family pyrin domain containing 3 (NLRP3) inflammasome signaling pathway. A and B, the expression of caspase-1, NLRP3, and interleukin-1β (IL-1β) proteins in cardiac tissues was detected by IHC. n = 5; * P < 0.05, ** P < 0.01, *** P< 0.001.

**Figure 5.**
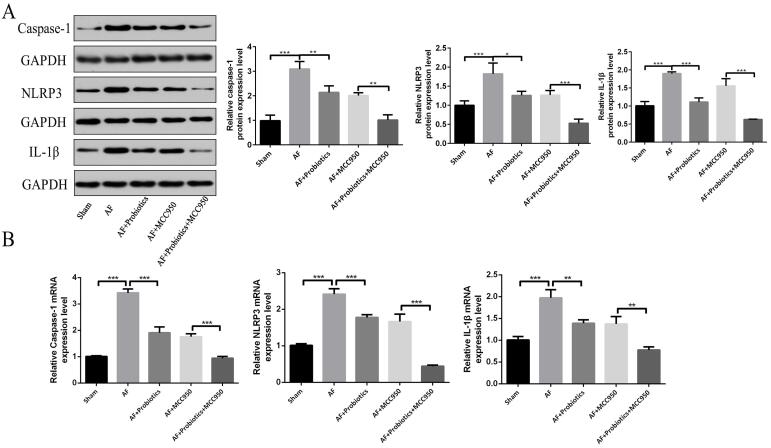
Probiotics inhibited the activation of the NOD-like receptor family pyrin domain containing 3 (NLRP3) inflammasome signaling pathway in cardiac tissues. The expression of caspase-1, NLRP3, and interleukin-1β (IL-1β) was detected by Western blot (A) and real-time quantitative polymerase chain reaction (RT-qPCR) (B). n = 5; * P < 0.05, ** P < 0.01, *** P< 0.001.

Next, we investigated the expression of genes and proteins associated with the NLRP3 inflammasome signaling pathway in AF. IHC analysis revealed that, compared with the Sham group, the expression levels of NLRP3, caspase-1, and IL-1β in the cardiac tissue of AF model rats were significantly elevated, suggesting that inflammation was activated during AF onset. Compared with the AF group, probiotics significantly attenuated the expression of NLRP3, caspase-1, and IL-1β in the cardiac tissue of AF rats. Similarly, the NLRP3 inhibitor MCC950 also markedly suppressed the expression of these proteins. Notably, the combination of probiotics and MCC950 exerted an even stronger inhibitory effect than either treatment alone (P < 0.05 vs. AF+MCC950; P < 0.01 vs. AF+Probiotics), indicating a synergistic interaction between probiotics and pharmacological NLRP3 inhibition (Figure 4A and B). This enhanced effect may be attributed to the dual mechanism of action: Probiotics restoring gut microbiota homeostasis and increasing SCFA production, while MCC950 directly blocks NLRP3 activation, together achieving more comprehensive suppression of the inflammatory cascade. Consistent with the IHC results, Western blot and RT-qPCR analyses further confirmed these findings (Figure 5A and B). Collectively, these data indicate that probiotics downregulate the NLRP3 inflammasome signaling pathway.

### 4.4. Probiotics Improved Atrial Fibrillation by Regulating Intestinal Flora Imbalance and the NOD-Like Receptor Family Pyrin Domain Containing 3 Inflammasome Signaling Pathway in Rats

**Figure 6.**
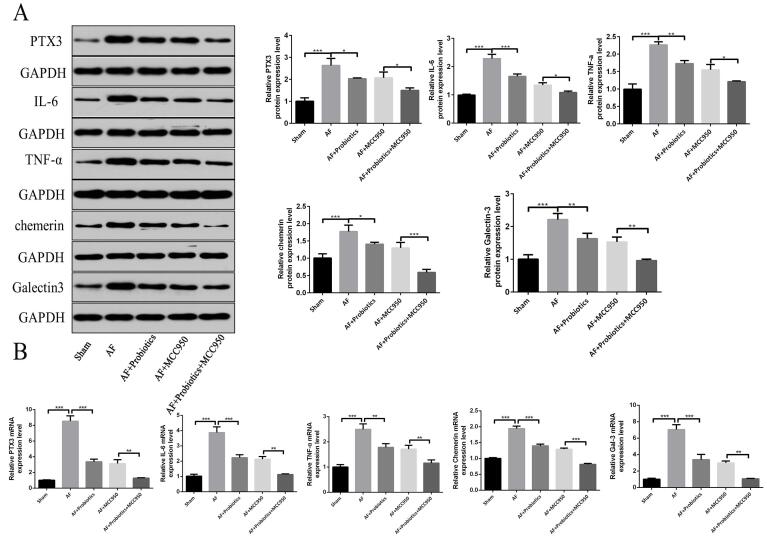
Probiotics improved atrial fibrillation (AF) by regulating intestinal flora imbalance and the NOD-like receptor family pyrin domain containing 3 (NLRP3) inflammasome signaling pathway in rats. The expression of AF biomarkers pentraxin 3 (PTX3), interleukin-6 (IL-6), tumor necrosis factor-alpha (TNF-α), chemerin, and Gal-3 was detected by Western blot (A) and real-time quantitative polymerase chain reaction (RT-qPCR) (B). n = 5; * P < 0.05, ** P < 0.01, *** P < 0.001.

Pentraxin 3, IL-6, TNF-α, chemerin, and Gal-3 in atrial tissue are potential biomarkers of the pathological process of AF. Therefore, we examined the effects of probiotics on the expression levels of these proteins and genes. Our results demonstrated that, compared with the Sham group, the expression of PTX3, IL-6, TNF-α, chemerin, and Gal-3 was significantly upregulated in the AF group, and probiotics treatment markedly reversed this upregulation. Additionally, compared with the AF group, the expression of these markers was significantly reduced in the AF+MCC950 group. Notably, the combination of probiotics and MCC950 exerted an even stronger inhibitory effect than MCC950 alone (P < 0.05 for all markers), with expression levels approaching those of the Sham group (Figure 6A and B). This further supports the synergistic benefit of combining gut microbiota modulation with direct NLRP3 inhibition in attenuating AF-associated pathology. These findings suggest that probiotics alleviated AF in rats by modulating intestinal flora imbalance and suppressing the NLRP3 signaling pathway.

## 5. Discussion

Atrial fibrillation remains a prevalent arrhythmia with complex pathogenesis, and recent evidence underscores the significance of the gut-heart axis in its development. While dysbiosis of the gut microbiota and NLRP3 inflammasome-driven inflammation have been independently implicated in AF, the therapeutic potential of probiotics and the mechanistic link involving microbial metabolites remain largely unexplored. Our study demonstrates that probiotic intervention alleviates AF pathology by restoring gut microbiota homeostasis, enhancing SCFA production, and subsequently suppressing the cardiac NLRP3 inflammasome pathway, thereby reducing the expression of key AF biomarkers.

Our findings reinforce the close association between gut dysbiosis and AF. Consistent with clinical observations in AF patients (17, 18), our rat model exhibited a significant reduction in microbial diversity and a marked depletion of beneficial genera, such as Lactobacillus. Probiotic administration effectively rectified these imbalances, confirming the capacity of probiotics to restore a healthy microbial ecosystem and establishing the foundation for the observed therapeutic effects. Similar gut microbiota alterations have been reported in other cardiovascular conditions, supporting the concept of a gut-heart axis (7, 9). Furthermore, recent large-scale metabolomics studies have identified phenylacetylglutamine (PAGln), a gut microbiota-dependent metabolite derived from dietary phenylalanine via the microbial porA gene, which promotes platelet hyperreactivity and thrombotic potential through adrenergic receptor signaling and is independently associated with increased risk of major adverse cardiovascular events (19). This finding further underscores the critical role of gut microbial metabolites in cardiovascular disease pathogenesis and aligns with our observation that probiotic-induced restoration of gut microbiota can mitigate AF-related pathology.

Crucially, we moved beyond compositional changes to investigate a functional outcome: microbial metabolism. The observed reduction in SCFA-producing bacteria in AF rats translated into significantly lower systemic levels of acetic, propionic, and butyric acids. Probiotics restored SCFA production, positioning these metabolites as key mediators between the remodeled gut microbiota and the host's cardiovascular system. This finding provides a plausible functional link through which gut dysbiosis may remotely influence cardiac inflammation.

Our results align with previous studies showing that SCFAs exert anti-inflammatory effects via GPR43 and HDAC inhibition (20). Collectively, a growing body of evidence has established gut microbiota-derived SCFAs as critical functional mediators that translate dietary modulation of microbial ecology into systemic cardiometabolic benefits, positioning them at the nexus of the gut-heart axis (21).

However, our study design does not establish a direct causal link between SCFAs and NLRP3 inhibition in the heart. While it is plausible that SCFAs act through G-protein coupled receptors (e.g., GPR43) or inhibit histone deacetylases to mediate these effects (20), future studies using SCFA receptor knockout models are needed to validate this hypothesis. Nevertheless, the central mechanistic insight of our work is the correlation between probiotic-induced increases in SCFAs and the suppression of the NLRP3 inflammasome pathway in cardiac tissue. The downregulation of NLRP3, caspase-1, and IL-1β, coupled with the concomitant reduction in AF biomarkers, strongly suggests that probiotic benefits are mediated, at least in part, through quenching this inflammatory cascade. This is consistent with the known immunomodulatory role of probiotics in suppressing NLRP3 activation in other inflammatory diseases, such as ulcerative colitis (15), IgA nephropathy (16), and even viral infections (22).

When comparing the effects of MCC950 alone versus combined probiotics+MCC950, we observed that the combination therapy yielded significantly greater improvements in microbial diversity, SCFA levels, and inflammatory markers. This suggests a synergistic mechanism: probiotics restore gut ecology and enhance SCFA production, while MCC950 directly inhibits NLRP3, together providing a dual benefit. These findings are in line with a recent study by Tan et al. (16), which reported similar synergistic effects in IgA nephropathy.

A key consideration in interpreting our findings is the experimental model. We employed the ACH/CaCl₂-induced AF model, which is well-established for studying acute arrhythmogenesis and inflammatory triggers 23. While it effectively allowed us to probe the gut-heart axis, it may not fully recapitulate the chronic structural remodeling seen in persistent AF. Therefore, our findings primarily illustrate the potent anti-inflammatory and biomarker-modulating effects of probiotics. Furthermore, the relatively small sample size (n = 5 per group) suggests that our results, while statistically significant, should be validated in larger cohorts. This profile is particularly relevant for clinical conditions driven by acute inflammation, such as postoperative AF (POAF). Consequently, our research provides a mechanistic rationale for exploring perioperative probiotic supplementation as a strategy to mitigate POAF risk in cardiac surgery patients.

In conclusion, our study delineates a pathway by which probiotics mitigate AF-associated pathology: through modulation of the gut microbiota, enhancement of SCFA production, and subsequent suppression of the NLRP3 inflammasome. Although the precise molecular link between SCFAs and cardiac NLRP3 requires further elucidation, our work solidifies the gut-heart axis as a legitimate therapeutic target and positions probiotics as a promising, non-invasive adjunct for AF management, meriting further clinical investigation.

### 5.1. Conclusions

The findings demonstrate that probiotics exert effects in regulating gut microbiota and suppressing the NLRP3 pathway, leading to the reduction of AF biomarkers and revealing a novel therapeutic mechanism for AF.

## Data Availability

The 16S rRNA sequencing data presented in this study are openly available in the NCBI Sequence Read Archive (SRA) at [https://www.ncbi.nlm.nih.gov/sra/PRJNA1364835], reference number PRJNA1364835. All other datasets generated and analyzed during the current study are available from the corresponding author upon reasonable request. The data are not publicly available due to the large volume of raw data and ongoing analyses.
